# Supplementary material for: The Second Highest Prevalence of Celiac Disease Worldwide: Genetic and Metabolic Insights in Southern Brazilian Mennonites
Source: Genes (Basel). 2023 Apr 30;14(5):1026. doi: 10.3390/genes14051026 (PMC10218569; doi:10.3390/genes14051026)
Supplement: Supplementary file 1 [file genes-14-01026-s001.zip › genes-2202237-supplementary.pdf]

Article

The Second Highest Prevalence of Celiac Disease Worldwide: Genetic and Metabolic Insights in Southern Brazilian Mennonites

Luana Caroline Oliveira [1,2], Amanda Coelho Dornelles [1], Renato Mitsunori Nisihara [3], Estevan Rafael Dutra Bruginski [4], Priscila Ianzen dos Santos [1,5], Gabriel Adelman Cipolla [1,2], Stefanie Epp Boschmann [3,5], Iara José de Messias-Reason [3,5], Francinete Ramos Campos [4], Maria Luiza Petzl-Erler [1,2] and Angelica Beate Winter Boldt [1,2,*]

[1] Laboratory of Human Molecular Genetics, Department of Genetics, Federal University of Paraná (UFPR), Centro Politécnico, Jardim das Américas, Curitiba 81531-990, Paraná, Brazil
[2] Postgraduate Program in Genetics, Department of Genetics, Federal University of Paraná (UFPR), Centro Politécnico, Jardim das Américas, Curitiba 81531-990, Paraná, Brazil
[3] Laboratory of Molecular Immunopathology, Department of Clinical Pathology, Clinical Hospital, Federal University of Paraná (UFPR), Rua General Carneiro, 181 Prédio Central, 11° Andar, Alto da Glória, Curitiba, 80060-240, Paraná, Brazil
[4] Postgraduate Program in Pharmaceutical Sciences, Laboratory of Bioscience and Mass Spectrometry, Department of Pharmacy, Federal University of Paraná (UFPR), Av. Pref. Lothário Meissner, 632, Jardim Botânico, Curitiba 80210-170, Paraná, Brazil
[5] Postgraduate Program in Internal Medicine, Federal University of Paraná (UFPR), Rua General Carneiro, 181 Prédio Central, 11° Andar, Alto da Glória, Curitiba 80060-240, Paraná, Brazil
* Correspondence: angelicaboldt@gmail.com; Tel.: +55-41-99910-4470

Supplementary Table S1 – Serological screening in the Mennonite population.

Test	CON +	CON -	N	CWI +	CWI -	N	CTB +	CTB -	N	N +	N -	Total
DGP	0	80	80	0	58	58	0	88	88	0	226	226
tTG	1	26	27	0	67	67	1	63	64	2	156	158
EMA	6	87	93	4	78	82	0	17	17	10	184	192
Previous	0	0	0	3*	0	3*	6	19	25	9	17	28
Total	7	193	200	7	203	210	7	187	194	21	583	604

DGP - anti-deamidated gliadin-related peptide, tTG - anti-tissue transglutaminase, EMA - human endomysial IgA antibody screening, CON – Colônia Nova, CWI – Colônia Witmarsum, CTB – Curitiba, N – number of individuals. * screened as EMA negative, but had been previously diagnosed for CD and were already in GFD, at the time of participant recruitment.

Supplementary Table S2 – PCR reagents for the identification of CD-associated HLA-DQ allele carriers.

	*DQA1*05* and *DQA1*03*			*DQB1*02*		*DQB1*03:02*	
	**[Initial]**	**[Final]**	**Volume (µL)**	**[Final]**	**Volume (µL)**	**[Final]**	**Volume (µL)**
***HLA* forward primer (ng/µL)**	10	0.15	0.25	0.18	0.3	0.2	0.25
***HLA* reverse primer (ng/µL)**	10	0.15	0.25	0.18	0.3	0.2	0.25
***GALC* forward primer (ng/µL)**	10	0.075	0.125	0.075	0.125	0.1	0.125
***GALC* reverse primer (ng/µL)**	10	0.075	0.125	0.075	0.125	0.1	0.125
**Genomic DNA (ng/µL)**	20	0.3	1	0.3	1	0.4	1
**GoTaq® RealTime qPCR master mix (x)**	2	7.5	2.5	7.5	2.5	-	-
**KAPA SYBR® FAST qPCR master mix (x)**	2	-	-	-	-	16	4
**DMSO (%)**	5	-	-	-	-	40	0.25
**H_2O (µL)**	-	-	1.75	-	1.65	-	2
**Total volume per reaction**			6		6		8

Within brackets: concentration. HLA – Human Leukocyte Antigen, GALC – galactosylceramidase, qPCR – quantitative polymerase chain reaction, DMSO – dimethyl sulfoxide

Supplementary Table S3 – PCR conditions for the identification of CD-associated HLA-DQ allele carriers.

	DQA1*05* and *DQA1*03			***DQB1*02***			***DQB1*03:02***		
**Parameter**	**Temperature (°C)**	**Time**	**Cycles**	**Temperature (°C)**	**Time**	**Cycles**	**Temperature (°C)**	**Time**	**Cycles**
**Initial denaturation**	95	10 min	1	95	10 min	1	94	5 min	1
	95	15 sec		95	15 sec		94	20 sec	
**Amplification**	60	60 sec	32	55	60 sec	32	60	20 sec	66
	-	-		-	-		72	40 sec	
**Melt curve – step 1**	95	15 sec		95	15 sec		95	15 sec	
**Melt curve – step 2**	60	60 sec	1	60	60 sec	1	60	60 sec	1
**Melt curve – step 3**	95	15 sec		95	15 sec		95	15 sec	

Supplementary Table S4: HLA-DQ2 and HLA-DQ8 carrier frequencies estimated for the Brazilians and the Belgians, compared with the Mennonite population.

Population	DQ2 *DQA1*05-DQB1*02*	Combined Frequency	Homozygote Frequency	Heterozygote Frequency	Carrier Frequency	Carriers (N)	Fisher's exact P value
**Brazil**	cis	0.0913	0.0083	0.1659	0.1742	112	
**(N=641)**	trans	0.0259	0.0007	0.0504	0.0510	33	
	total	0.1171	0.0090	0.2162	0.2252	144	0.0075
**Belgium**	cis	0.1410	0.0199	0.2422	0.2621	187	
**(N=715)**	trans	0.0126	0.0002	0.0250	0.0251	18	
	total	0.1536	0.0200	0.2672	0.2872	205	ns
**Mennonites (N=383)**	total	nd	nd	nd	0.3003	115	
	**DQ8** ***DQA1*03-DQB1*03:02***						
**Brazil**	cis	0.0835	0.0070	0.1530	0.1600	103	
**(N=641)**	trans	0.0005	0	0.0011	0.0011	0	
	total	0.0840	0.0070	0.1541	0.1610	103	0.0004
**Belgium* (N=715)**	cis	0.0730	0.0053	0.1353	0.1407	101	
**Mennonites (N=383)**	total	nd	nd	nd	0.2507	96	0.00001

The frequency of HLA-DQ2.5 and HLA-DQ8 in cis combinations was obtained by summing the frequencies of corresponding HLA haplotypes reported in the Allele Frequency Net Database (http://www.allelefrequencies.net/). To obtain the frequency of trans combinations, the sum of corresponding frequencies for *HLA*DQA1* alleles (*DQA1*05:01 + DQA1*05:03 + DQA1*05:05 for HLA-DQ2.5, DQA1*03:01, DQA1*03:02* and *DQA1*03:03* for HLA-DQ8) and HLA*DQB1 alleles (*DQB1*02:01 + DQB1*02:02 + DQB1*02:03* for HLA-DQ2.5, *DQB1*03:02* for HLA-DQ8) reported in the Allele Frequency Net Database was subtracted from the

sum of in cis frequency combinations. *DQA1*05* and *DQB1*02* resulting frequencies were multiplied to obtain HLA-DQ2.5 trans combinations, as well as *DQA1*03* and *DQB1*03:02*, to obtain HLA-DQ8 trans combinations. The resulting cis and trans frequencies were summed to obtain the HLA-DQ2.5 and HLA-DQ8 combined frequencies. Homozygote and heterozygote carrier frequencies were obtained from the cis, trans and total HLA combination frequencies, assuming Hardy and Weinberg equilibrium. Carrier frequencies were estimated through the sum of homozygote and heterozygote carrier frequencies. Absolute numbers were compared with Fisher's exact test (two-sided P value), between Brazilians and Mennonites, and between Belgians and Mennonites.

* there were no HLA-DQ8 "trans" combinations for the Belgian population, all *DQB1*03:02* alleles were found in absolute linkage disequilibrium with *DQA1*03* alleles.

nd: not determined (homozygotes were not differentiated from heterozygotes in our genotyping approach), ns: not significant.
